# Author Correction: Amantadine inhibits known and novel ion channels encoded by SARS-CoV-2 in vitro

**DOI:** 10.1038/s42003-021-02940-2

**Published:** 2021-12-10

**Authors:** Trine Lisberg Toft-Bertelsen, Mads Gravers Jeppesen, Eva Tzortzini, Kai Xue, Karin Giller, Stefan Becker, Amer Mujezinovic, Bo Hjorth Bentzen, Loren B. Andreas, Antonios Kolocouris, Thomas Nitschke Kledal, Mette Marie Rosenkilde

**Affiliations:** 1 Department of Biomedical Sciences, Faculty of Health and Medical Sciences, University of Copenhagen, Copenhagen, Denmark; 2 Department of Neuroscience, Faculty of Health and Medical Sciences, University of Copenhagen, Copenhagen, Denmark; 3 Synklino ApS, Charlottenlund, Denmark; 4 Laboratory of Medicinal Chemistry, Section of Pharmaceutical Chemistry, Department of Pharmacy, National and Kapodistrian University of Athens, Panepistimioupolis-Zografou, Athens, Greece; 5 Department of NMR-based structural biology, Max Planck Institute for Biophysical Chemistry, Göttingen, Germany

**Keywords:** Molecular medicine, Viral infection

Correction to: *Communications Biology* 10.1038/s42003-021-02866-9; published online 01 December 2021.

In the original version of the Article, the title did not accurate reflect the main findings of the study. In addition, the final sentence of the Abstract and parts of the text referring to references 3–5 were found to be misleading. The following corrections have been made in the PDF and HTML versions of the Article:

Original Title: Amantadine has potential for the treatment of COVID-19 because it inhibits known and novel ion channels encoded by SARS-CoV-2.

Corrected Title: Amantadine inhibits known and novel ion channels encoded by SARS-CoV-2 in vitro.

Abstract: The final sentence of the abstract was removed (“We therefore propose amantadine as a novel, cheap, readily available and effective way to treat COVID-19”).

Introduction, first paragraph

Original text: A recent retrospective cohort study described an apparent increase in survival in coronavirus disease 2019 (COVID-19) patients treated with amantadine^3^. Importantly, a study based on self-reported COVID-19 disease among users of amantadine for neurological diseases^4^ and a small-scale treatment of COVID-19 patients with amantadine^5^ both supported this observation.

Corrected text: A recent retrospective cohort study evaluating amantadine amongst other antivirals did not find any significant benefit of amantadine in the treatment of coronavirus disease 2019 (COVID-19) patients^3^. However, a study based on self-reported COVID-19 disease among users of amantadine for neurological diseases^4^ and a small-scale treatment of COVID-19 patients with amantadine^5^ suggested a positive impact.

Discussion, second to last paragraph

In the following sentence, the word “use” was incorrect and has been replaced with “further test”:

“We propose to use amantadine as a novel and effective way to treat COVID-19 through its ability to inhibit known (Protein E) and novel (ORF10) ion channels.”

The following sentence has been removed entirely:

Importantly, in the clinic, an apparent protective effect of amantadine in COVID-19 patients has been reported in a retrospective cohort study in Mexico^3^ and in a small-scale treatment of 15 COVID-19 patients^5^.

