# Supplementary material for: Amantadine inhibits known and novel ion channels encoded by SARS-CoV-2 in vitro
Source: Commun Biol. 2021 Dec 1;4:1347. doi: 10.1038/s42003-021-02866-9 (PMC8636635; doi:10.1038/s42003-021-02866-9)
Supplement: Supplementary file 2 — Supplementary Information [file 42003_2021_2866_MOESM2_ESM.pdf]

Supplementary Information

Amantadine has potential for the treatment of COVID-19 because it inhibits known and novel ion channels encoded by SARS-CoV-2

Trine Lisberg Toft-Bertelsen[1,2#], Mads Gravers Jeppesen[1,3#], Eva Tzortzini,[4±] Kai Xue[5±], Karin Giller[5], Stefan Becker[5], Amer Mujezinovic[1], Bo Hjorth Bentzen[1], Loren Andreas[5], Antonios Kolocouris[4], Thomas Nitschke Kledal[3]* and Mette Marie Rosenkilde[1]*

[1] *Department of Biomedical Sciences, Faculty of Health and Medical Sciences, University of Copenhagen, Copenhagen, Denmark*
[2] *Department of Neuroscience, Faculty of Health and Medical Sciences, University of Copenhagen, Copenhagen, Denmark*
[3] *Synklino ApS, Charlottenlund, Denmark*
[4] *Laboratory of Medicinal Chemistry, Section of Pharmaceutical Chemistry, Department of Pharmacy, National and Kapodistrian University of Athens, Panepistimioupolis-Zografou, Athens, Greece*
[5] *Department of NMR-based structural biology, Max Planck Institute for Biophysical Chemistry, Göttingen, Germany*

[#, ±] *These authors contributed equally to this work*

Correspondence:
Mette Marie Rosenkilde, University of Copenhagen, Faculty of Health and Medical Sciences, Department of Biomedical Sciences Blegdamsvej 3, DK-2200 Copenhagen, Denmark; Phone +45 30 60 46 08; E-mail: rosenkilde@sund.ku.dk, ORCiD 0000-0001-9600-3254

Thomas Nitschke Kledal, Synklino ApS, Raadhusvej 13, DK-2920 Charlottenlund, Denmark; Phone +45 20 12 16 56; E-mail: tnk@synklino.com, ORCiD 0000-0002-6320-4452

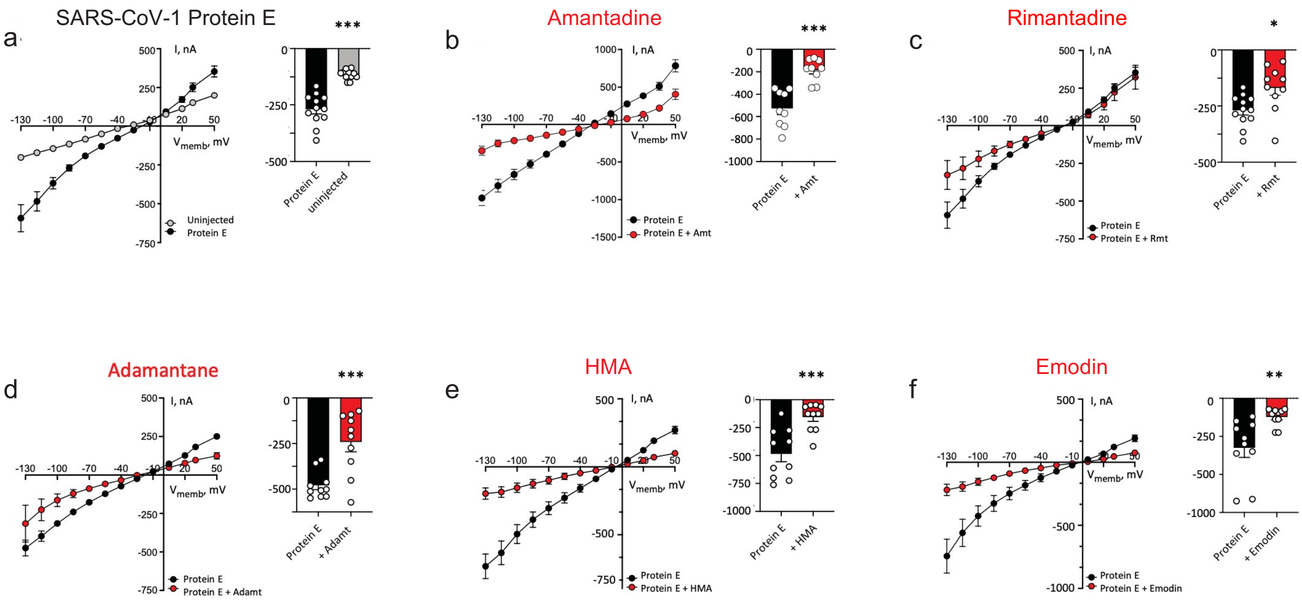

Supplementary Fig. 1. Impact of drugs on the ion channel function of Protein E from SARS-CoV-1││ a, Summarized and averaged I/V relations in SARS-CoV-1 Protein E-expressing oocytes revealed significantly different current activity compared to control (uninjected) oocytes. **Inset**, The current activity at -85mV in SARS-CoV-1 Protein E-expressing oocytes normalized to that of uninjected oocytes. **b-f**, The drugs (10 μM) amantadine (**b**), rimantadine (**c**), adamantane (**d**), HMA (**e**) and emodin (**f**) inhibited Protein E from SARS-CoV-1. **Insets (b-f)**, current activity at -85mV with treatment of the specific drugs normalized to the current activity obtained without treatment. Statistical significance was determined with unpaired Student *t* test, * P < 0.05; **P < 0.01; ***P < 0.001, of n=5 biologically independent experiments.

a

```
SARS-CoV-2 ProtE    MYSFVSEETGTLIVNSVLLFLAFVVFLLVTLAILTALRLCAYCCNIVNVSLVKPSFYVYSRVKNLNSSRVPDLLV

SARS-CoV-2 ORF7b              MIELSLIDFYLCFLAFLLFLVLIMLIIFWFSLELQDHNETCHA

TGEV ProtE                              MTFPRALTVIDDNGMVISIIFWFLLIIILILLSIALLNIIKLCMVCCNLGRTVIIVPAQHAYDAYKNFMRIKAYNPDGALLA
```

b

```
SARS-CoV-1 ProtE    MYSFVSEETGTLIVNSVLLFLAFVVFLLVTLAILTALRLCAYCCNIVNVSLVKPTVYVYSRVKNLNSSEGVPDLLV

SARS-CoV-1 ORF7b              MNELTLIDFYLCFLAFLLFLVLIMLIIFWFSLEIQDLEEPCTKV

SARS-CoV-1 Orf8a                     MKLLIVLTCISLCSCICTVVQRCASNKPHVLEDPCKVQ
```

c

```
SARS-CoV-1 Protein 3a    MDLFMRFFTLRSITAQPVKIDNASPASTVHATATIPLQASLPFGWLVIGVAFLAVFQSAT    60
SARS-CoV-2 Protein 3a    MDLFMRIFTIGTVTLKQGEIKDATPSDFVRATATIPIQASLPFGWLIVGVALLAVFQSAS    60
                         ******:**:  ::*  :   :*.:*:*:.  *:******:*********::***:*******:

SARS-CoV-1 Protein 3a    KIIALNKRWQLALYKGFQFICNLLLLFVTIYSHLLLVAAGMEAQFLYLYALIYFLQCINA    120
SARS-CoV-2 Protein 3a    KIITLKKRWQLALSKGVHFVCNLLLLFVTVYSHLLLVAAGLEAPFLYLYALVYFLQSINF    120
                         ***:*:******* **.:*:*********:**********:** *******:****.**

SARS-CoV-1 Protein 3a    CRIIMRCWLCWKCKSKNPLLYDANYFVCWHTHNYDYCIPYNSVTDTIVVTEGDGISTPKL    180
SARS-CoV-2 Protein 3a    VRIIMRLWLCWKCRSKNPLLYDANYFLCWHTNCYDYCIPYNSVTSSIVITSGDGTTSPIS    180
                          ***** ******:************:****: ***********.:**:*.*** ::*

SARS-CoV-1 Protein 3a    KEDYQIGGYSEDRHSGVKDYVVVHGYFTEVYYQLESTQITTDTGIENATFFIFNKLVKDP    240
SARS-CoV-2 Protein 3a    EHDYQIGGYTEKWESGVKDCVVLHSYFTSDYYQLYSTQLSTDTGVEHVTFFIYNKIVDEP    240
                         :.*******:*. .***** **:*.***. **** ***::****:*:.****:**:*.:*

SARS-CoV-1 Protein 3a    P-NVQIHTIDGSSGVANPAMDPIYDEPTTTTSVPL    274
SARS-CoV-2 Protein 3a    EEHVQIHTIDGSSGVVNPVMEPIYDEPTTTTSVPL    275
                           :************.**.*:**************
```

Supplementary Fig. 2. **Amino acid sequences of viroporins from SARS-CoV-1 and - 2 │ a**, Homology between SARS-CoV-2 Protein E and ORF7b (grey highlight). The identified sequence is located in the predicted transmembrane region of the proteins (underlined). A region of homology (yellow highlight) also exists between the C-terminal part of the transmembrane region of ORF7b and that of porcine transmissible gastroenteritis coronavirus (TGEV) Protein E. **b**, The sequence homology between Protein E and ORF7b is also present in SARS-CoV-1. There is an additional homology (green highlight) between SARS-CoV-1 ORF7b and Orf8a in their C-termini. **c,** Sequence alignment of Protein 3a from SARS-CoV-1 and -2. The predicted transmembrane region of SARS-CoV-2 Protein 3a is underlined.

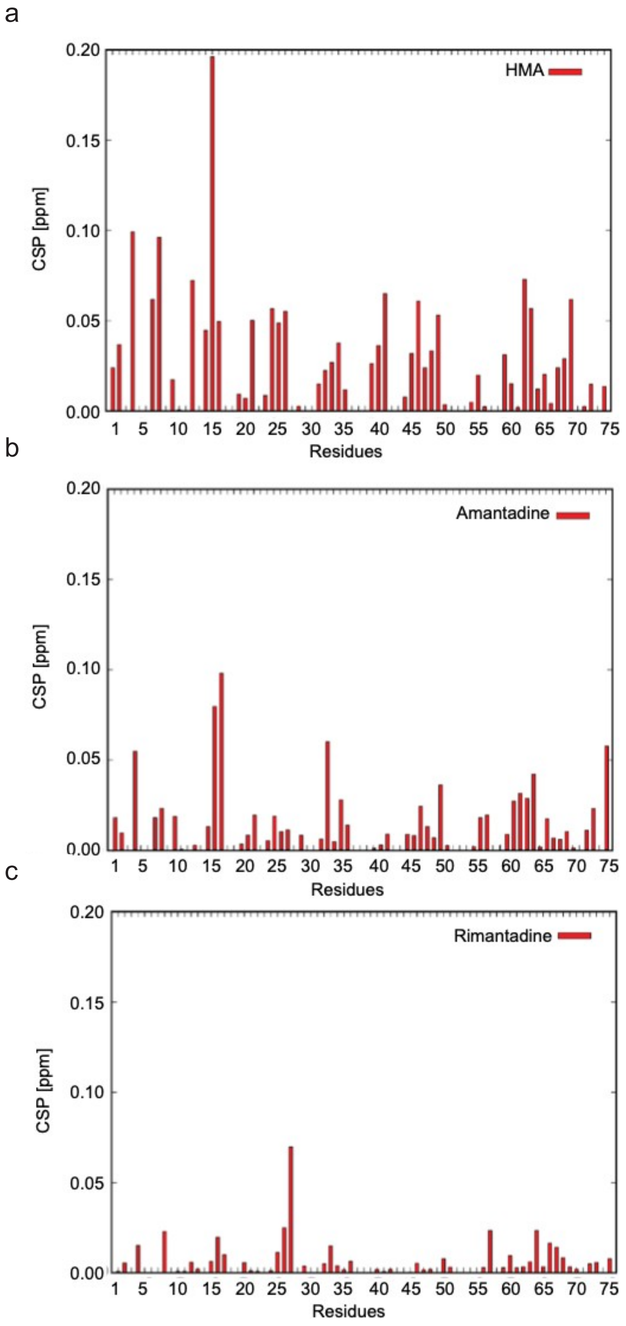

Supplementary Fig. 3. CSPs on ^{15}N resonances of protein E after addition of HMA, amantadine or rimantadine at 2mM │ Per-residue CSP values for **a**, HMA, **b**, amantadine and **c**, rimantadine were calculated according to √((δ_H^2+0.14*δ_N^2)/2). Measurements were recorded at 50 °C using a Bruker Prodigy probe on a 600 MHz instrument.

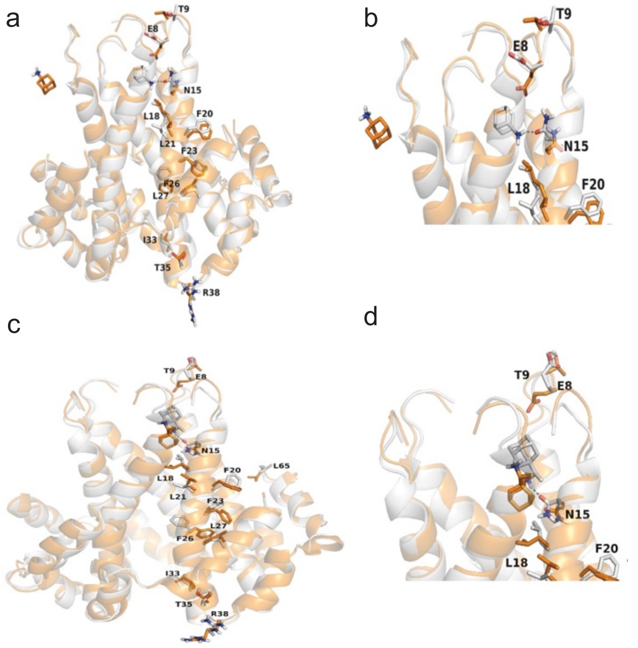

Supplementary Fig. 4. Frames of Protein E (aa 8-65) in complex with amantadine or rimantadine │ The complexes are shown at 0 ns (ligand carbons and Protein E in grey) and at 100 ns (ligand carbons in orange) from restrained 100 ns-MD simulations with OPLS2005 and an applied force constant of 2 kcal mol $Å^{-2}$ to the Ca atoms of Protein E. **a and b**, Protein E in complex with amantadine using as starting structure a docking pose for amantadine in the inward orientation; the drug escapes from the protein during the simulation (b is a zoom in of a). **c and d,** Protein E in complex with rimantadine, using as starting structure a docking pose for rimantadine in the inward orientation where the simulation resulted in a flipping of the drug in the outward orientation; starting another 100-ns MD simulation with the outward orientation the drug also rotates by 180° to the inward orientation. This fluctuation suggests an unstable complex (d is a zoom in of c). Ligand and amino acid residues (shown in only one of the five α-helices for clarity) are shown as sticks. The PDB ID 5X29[16] was used as starting structure of Protein E (aa 8-65) in the Protein E-amantadine complex.

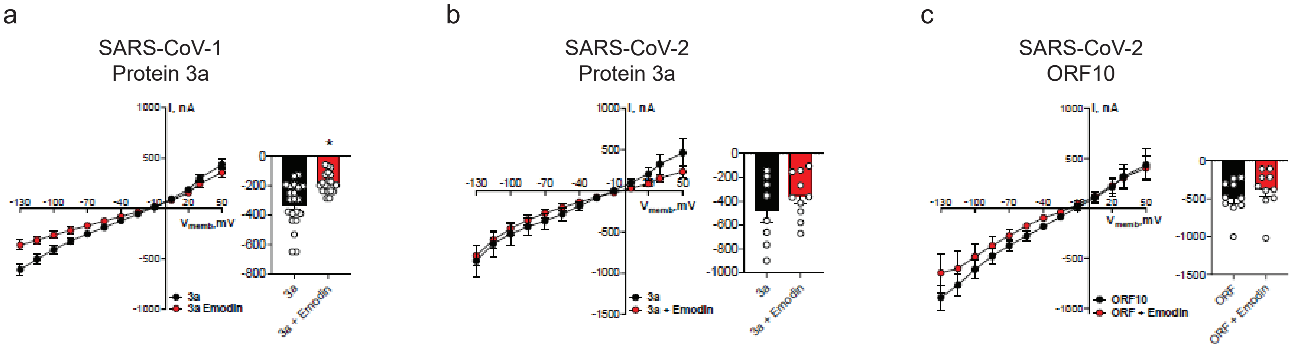

Supplementary Fig. 5. The effect of Emodin on Protein 3a and ORF10│a, Summarized and averaged I/V relations in SARS-CoV-1 Protein 3a-expressing oocytes revealed inhibitory effect of emodin on current activity. **Inset**, current activity at -85mV with treatment of emodin (10 μM) normalized to the current activity obtained without treatment. **b-c**, Summarized and averaged I/V relations in SARS-CoV-2 Protein 3a (**b**) and ORF10 (**c**) expressing oocytes. No inhibitory effect of emodin on current activity was found. **Inset**, current activity at -85mV with treatment of emodin normalized to the current activity obtained without treatment. Statistical significance was determined with unpaired Student *t* test, * $P < 0.05$, of n=4 biologically independent experiments.

Drug	Protein 3a	ORF7b	ORF10
Amantadine	no effect	no effect	*reduction*
Rimantadine	no effect	no effect	no effect
Adamantane	no effect	no effect	no effect
Emodin	no effect	*reduction*	no effect
Pyronin B	no effect	no effect	no effect
Xanthene	*reduction*	reduction	no effect
Pyronin Y	no effect	no effect	no effect

Supplementary Table 1. The effect of selected drugs on Protein 3a, ORF7b and ORF10 from SARS-CoV-2│Summarized effects of amantadine, rimantadine, adamantane, emodin, pyronin B, xanthene and pyronin Y on Protein 3a, ORF7b and ORF10 expressing oocytes. Inhibitory effect was found of amantadine (ORF10), emodin (ORF7b), and xanthene (Protein 3a and ORF7b) on current activity (reduction marked by italic letters). The remaining four drugs rimantadine, adamantine, pyronin B and pyronin Y displayed no reducing effect on current activity.

Supplementary Methods

Docking calculations of amantadine, rimantadine and HMA inside the Protein E pore | The structures of the compounds amantadine, rimantadine and HMA were built by means of Maestro[1]. Subsequently, these were minimized by employing the Macromodel 9.6[1], the MMFF94 force field[2], the conjugate gradient method and a distance-dependent dielectric constant until a convergence value of 0.0001 kJ Å^{-1} mol^{-1} was reached. The docking calculations of the energy minimized ligand structures to the Protein E (aa 8-65) were performed with the Glide program[3] and Glide XP scoring function which docks ligands flexibly[3,4] in a 20 Å radius from the reference docking pose. We obtained both the outward and inward orientation in docking solutions with the outward orientation being favored.

MD simulations | For the MD simulations of protein-drug complexes the particle mesh Ewald method (PME)[5] was applied to calculate the long-range electrostatic interactions with a grid spacing of 0.8 Å. Van der Waals and short-range electrostatic interactions were smoothly truncated at 12 Å. The Nosé-Hoover thermostat[6] was utilized to maintain a constant temperature in all simulations, and the Martyna-Tobias-Klein method[6] was used to control the pressure. The equations of motion were integrated using the multistep RESPA integrator[7] with an inner time step of 2 fs for bonded interactions and non-bonded interactions within a cutoff of 12 Å. An outer time step of 6.0 fs was used for non-bonded interactions beyond the cut-off. Each system was equilibrated using a modification of the default protocol provided in Desmond. The modification of the protocol consists of a series of restrained minimizations and MD simulations designed to relax the system, while not deviating substantially from the initial coordinates. First, two rounds of steepest descent minimization were performed using a maximum of 2000 steps and harmonic restraints of 50 kcal mol Å^{-2} applied on all solute atoms, followed by 10000 steps of minimization without restraints. The first simulation was run for 200 ps at a temperature of 10 K in the NVT ensemble with solute heavy atoms restrained by a force constant of 50 kcal mol Å^{-2}. The temperature was then raised during a 200 ps MD simulation to 310 K in the NVT ensemble with the force constant retained. The temperature of 310 K was used in MD simulations in order to ensure that the membrane state is above the main phase transition temperature of 271 K observed for POPC and 297 for DMPC bilayers[8]. The heating was then followed by equilibration simulations. First, two 1 ns stages of NPT equilibration were performed. In the first 1 ns stage, the heavy atoms of the system were restrained by applying a force constant of 10 kcal mol Å^{-2} for the harmonic constraints and in the second 1 ns stage the heavy atoms

of the protein-ligand complex were restrained by applying a force constant of 2 kcal mol Å^{-2} to equilibrate solvent and lipids.

In the production phase, the Protein E-drug complexes systems were simulated using a restraint of 2 kcal mol Å^{-2} to the Ca carbons of the Protein E in the NPT ensemble conditions for 100 ns. Within this simulation time, the total energy and the RMSD of the protein's backbone Cα atoms reached a plateau, therefore the systems were considered equilibrated and suitable for statistical analysis. The visualization of produced trajectories was performed using the GUI of Maestro and the protein-ligand interaction analysis was done with the Simulation Interaction Diagram (SID) tool, available with Desmond. For hydrogen bond interactions, a distance of 2.5 Å between donor and acceptor heavy atoms, and an angle ≥ 120° between donor-hydrogen-acceptor atoms and ≥ 90° between hydrogen-acceptor-bonded atoms were considered. Non-specific hydrophobic contacts were identified when the side chain of a hydrophobic residue fell within 3.6 Å from a ligand's aromatic or aliphatic carbon, while π-π interactions were characterized by stacking of two aromatic groups face-to-face or face-to-edge. Water-mediated interactions were characterized by a distance of 2.7 Å between donor and acceptor atoms, as well as an angle ≥ 110° between donor-hydrogen-acceptor atoms and ≥ 80° between hydrogen-acceptor-bonded atoms.

Supplementary References

1. Schrödinger. LigPrep. *Schrödinger Release 2020-4: Schrödinger, LLC, New York, NY, 2020* (2020).
2. Halgren, T. A. Merck molecular force field. I. Basis, form, scope, parameterization, and performance of MMFF94. *Journal of Computational Chemistry* **17**, (1996).
3. Friesner, R. A. *et al.* Glide: A New Approach for Rapid, Accurate Docking and Scoring. 1. Method and Assessment of Docking Accuracy. *Journal of Medicinal Chemistry* **47**, (2004).
4. Friesner, R. A. *et al.* Extra precision glide: Docking and scoring incorporating a model of hydrophobic enclosure for protein-ligand complexes. *Journal of Medicinal Chemistry* **49**, (2006).
5. Darden, T., York, D. & Pedersen, L. Particle mesh Ewald: An N·log(N) method for Ewald sums in large systems. *The Journal of Chemical Physics* **98**, (1993).
6. Martyna, G. J., Tobias, D. J. & Klein, M. L. Constant pressure molecular dynamics algorithms. *The Journal of Chemical Physics* **101**, (1994).
7. Humphreys, D. D., Friesner, R. A. & Berne, B. J. A multiple-time-step Molecular Dynamics algorithm for macromolecules. *Journal of Physical Chemistry* **98**, (1994).
8. Koynova, R. & Caffrey, M. Phases and phase transitions of the phosphatidylcholines. *Biochimica et Biophysica Acta - Reviews on Biomembranes* vol. 1376 (1998).
