# Supplementary material for: Amantadine inhibits known and novel ion channels encoded by SARS-CoV-2 in vitro
Source: Commun Biol. 2021 Dec 1;4:1347. doi: 10.1038/s42003-021-02866-9 (PMC8636635; doi:10.1038/s42003-021-02866-9)
Supplement: Supplementary file 3 — Description of Additional Supplementary Files [file 42003_2021_2866_MOESM3_ESM.pdf]

Description of Additional Supplementary Files

File name: Supplementary Data 1

Description: Source data for the graphs and charts.
